# Cardiovascular disease mortality: time series and spatial analysis, Brazil, 2003-2023

**DOI:** 10.1590/S2237-96222026v35e20250892.en

**Published:** 2026-03-15

**Authors:** Francisca Vaneska Lima Nascimento, Ítalo Rigoberto Cavalcante Andrade, Lara Lídia Ventura Damasceno, Thiago Santos Garces, Thereza Maria Magalhães Moreira, George Jó Bezerra Sousa, Maria Lúcia Duarte Pereira, Lúcia de Fátima da Silva

**Affiliations:** 1 Universidade Estadual do Ceará, Programa de Pós-Graduação Cuidados Clínicos em Enfermagem e Saúde, Fortaleza, CE, Brazil Universidade Estadual do Ceará Programa de Pós-Graduação Cuidados Clínicos em Enfermagem e Saúde Fortaleza CE Brazil; 2 Ministério da Saúde, Brasília, DF, Brazil Ministério da Saúde Brasília DF Brazil

**Keywords:** Cardiovascular Diseases, Mortality, Spatial Analysis, Time Factors, Secondary Data Analysis

## Abstract

**Objective:**

To describe the temporal and spatial distribution of cardiovascular disease mortality in Brazil from 2003 to 2023.

**Methods:**

A time series and spatial analysis study was conducted using secondary data from the Mortality Information System run by the Brazilian Unified Health System Department of Information Technology, including all deaths from circulatory system diseases. Sociodemographic characteristics were analyzed and standardized mortality rates per 100,000 inhabitants were calculated. The temporal trend was assessed using joinpoint regression, with estimated annual percentage change. The spatial analysis considered average municipal rates, both unadjusted and also smoothed by the local empirical Bayesian method, in addition to global and local spatial autocorrelation.

**Results:**

7,088,623 cardiovascular disease deaths were recorded in Brazil between 2003 and 2023, with predominance in males and higher mortality rates among the elderly and people with low levels of education. The highest rates were observed in the Southeast (190.14/100,000) and Southern (187.59/100,000) regions, while the Northern (APC 2003-2008 4.38; APC 2008-2023 2.33) and Northeast (APC 2003-2007 8.15; APC 2007-2023 1.11) regions showed an increasing trend over the period (p-value<0.001). Global Moran’s I (0.02; p-value<0.05) indicated weak but statistically significant positive spatial autocorrelation. Local analysis revealed clusters of high mortality concentrated mainly in municipalities of Mato Grosso and Goiás states, while areas of low mortality predominated in the Northern region and on the Northeast coast.

**Conclusion:**

Cardiovascular disease mortality in Brazil showed heterogeneous distribution in time and space.

**Ethical aspects**

This research used public domain anonymized databases.

## Introduction 

Cardiovascular diseases (CVD)s encompass a set of conditions that affect the heart and blood vessels, characterized by structural or functional alterations of the circulatory system. This group includes ischemic heart disease, heart failure, hypertensive diseases, cerebrovascular diseases, peripheral vascular diseases, among others. CVD is the leading cause of global mortality, and its occurrence is strongly influenced by modifiable risk factors such as hypertension, diabetes mellitus, dyslipidemia, smoking, physical inactivity, obesity, and inadequate diet, as well as social and environmental determinants, which include socioeconomic conditions, education, race/skin color, access to health services and territorial inequalities ^1^.

In 2019, CVDs were responsible for approximately 17 million deaths, 32% of global deaths, of which more than 80% occurred in low- and middle-income countries and almost a third were considered premature deaths (≤70 years) ^2^. In 2021, this number increased to 20 million deaths, a cumulative increase of almost 60% compared to levels observed since 1990 ^3^. In Brazil, the panorama follows this trend. Estimates indicate that, in 2021, the standardized mortality rate for coronary heart disease was 67/100,000 inhabitants and that cardiovascular causes accounted for 28% of deaths from non-communicable diseases, which contributed significantly to preventable mortality and years of potential life lost ^4^.

In economic terms and in terms of public health impact, studies from different periods help to compose this growing scenario. In Brazil in 2015, the cost of CVDs was estimated at R$ 37 billion, of which 61% referred to premature deaths and 39% corresponded to direct expenses and loss of productivity ^4^. In another national estimate, high sodium intake resulted, in 2017, in 46,651 preventable deaths and 575,172 adjusted years of life lost ^5^. Projections suggest that, between 2025 and 2050, cardiovascular mortality could increase by up to 73%, which would mean 35 million deaths, while adjusted years of life lost could increase by 54% ^3^.

Considering the epidemiological and economic relevance of CVDs, it becomes fundamental to identify how mortality indicators are distributed between different population strata, especially in the face of accelerated aging, regional inequalities, years of potential life lost, and associated direct and indirect costs. This understanding is essential to support public health decision-making and guide more effective policies tailored to territorial specificities. Use of secondary data allows for the continuous and comprehensive capture of cardiovascular mortality dynamics, which provides support for prevention, early detection, clinical management and appropriate resource allocation.

This design allows for an integrated assessment of the distribution of cardiovascular mortality in Brazil, enabling identification of more vulnerable regions and populations. This approach has been a priority since the adoption by the World Health Organization of the Global Action Plan for the Control of Noncommunicable Diseases (NCDs) 2013-2020 ^6^. Following this, the 2030 Agenda for Sustainable Development expanded this guideline, proposing a one-third reduction in premature mortality from these diseases by 2030. In Brazil, this international commitment has been incorporated through the Strategic Action Plan for Addressing NCDs in Brazil 2021-2030 ^7^.

The objective of this study was to describe the temporal and spatial distribution of CVD mortality in Brazil. 

## Methods 

Design

This is a time and spatial series analysis study, conducted using secondary data from the Mortality Information System (*Sistema de Informações sobre Mortalidade*, SIM), provided by the Brazilian Unified Health System Department of Information Technology (DATASUS), accessed via the Tabnet platform.

SIM is the official Brazilian death surveillance system, created to monitor the mortality profile in the country. It operates based on the death certificate, a document standardized at the national level and issued by health professionals. After completion, the death certificate is sent to the Municipal Health Departments, where it goes through stages of coding the underlying causes and contributing factors, according to the 10^th^ edition of the International Classification of Diseases (ICD-10). 

The data are then consolidated by the State Health Departments and the Ministry of Health, which performs review, validation and standardization routines for making the data publicly available ^8^.

Setting

The five macro-regions of Brazil (North, Northeast, South, Southeast, and Midwest) were taken as the geographic area of ​​interest for the time series, while the 5,570 Brazilian municipalities were taken to be the area of interest for the spatial analysis. 

Data source 

All deaths due to diseases of the circulatory system, listed in Chapter IX of the ICD-10, recorded between the 2003 and 2023 were considered. This Chapter IX covers the following list of morbidities: chronic rheumatic heart diseases (I05-I09); essential hypertension (I10); other hypertensive diseases (I11-I15); acute myocardial infarction (I21); other ischemic heart diseases (I20, I22-I25); pulmonary embolism (I26); conduction disorders and cardiac arrhythmias (I44-I49); heart failure (I50); other forms of heart disease (130-I43, I51); other circulatory diseases (I70-I99) ^9^. The databases used to build the results are available in the SciELO Data repository and are publicly accessible (https://doi.org/10.48331/SCIELODATA.MKZUPS) ^9^.

Statistical methods

The following variables were used for sociodemographic characterization: sex (male; female), age group in years (<4; 5-9; 10-14; 15-19; 20-29; 30-39; 40-49; 50-59; 60-69; 70-79; >80), race/skin color (White; Black; mixed-race; Asian; Indigenous) and schooling level (none; 1-3 years; 4-7 years; 8-11 years; ≥12). Demographic information to support the calculation of mortality rates was obtained from the "Demographic and Socioeconomic" section of the DATASUS portal, which provides population projections by sex, exact age or age group from 2000 to 2070. 

Mortality rates were standardized for each variable analyzed, thus allowing for more precise comparisons between different sociodemographic groups. The rates were calculated by taking the number of deaths in each sociodemographic stratum as the numerator and the respective populations belonging to the same demographic categories as the denominator, as provided by the population projections.

The time trend analyses were performed using the Joinpoint Regression Program, analyzing the entire period from 2003 to 2023, testing models with zero up to the maximum allowed number of inflection points. The traditional method based on Monte Carlo permutations was used, which allows p-values ​​associated with annual percentage change (APC) to be obtained. The APCs presented in this study were obtained based on the permutation test, accompanied by their respective 95% confidence intervals (95%CI) and p-values.

The first-order autoregressive component (AR=1) was considered in the model adjustment to minimize possible serial autocorrelation effects. This procedure aims to correct temporal dependencies between consecutive observations, which increases the precision of the estimates. The mortality rates were transformed using the natural logarithm (ln), according to the standard procedure of the software's log-linear model. This transformation allows for the interpretation of the slopes of the segments, as well as direct comparisons between different magnitudes of change over time. For this purpose, the death reporting year was defined as the independent variable, and the mortality rate as the dependent variable.

We used the death reporting year, as obtained using SIM/Tabnet, which represents the information consolidated by the Ministry of Health after the processing the death certificates. Although the year of occurrence of death is conceptually distinct, studies based on aggregated annual SIM data have shown minimal discrepancy between the two dates, especially in databases that have already been consolidated, so that this does not compromise temporal analysis.

The direction of the trend depended on the APC value and statistical significance. When the values ​​are positive and statistically significant (p-value<0.05), a rising trend is indicated. If they are negative and statistically significant, they point to a falling trend over time. If significance is not found (p-value>0.05), there is taken to be no relevant change in the period analyzed, which characterizes a stable or stationary trend ^10^.

In the spatial analysis, the average annual mortality rate adjusted for the resident population in the mid-range year was calculated for each municipality, in order to consider the differences in the demographic characteristics of the populations and reduce disparity bias, according to the following formula: average number of deaths per municipality (total deaths/number of years) divided by the resident population in the mid-range year, multiplied by the coefficient of 100,000 inhabitants.

In order to reduce the inherent instability of the unadjusted rates, rate smoothing was applied using the local empirical Bayesian method. This procedure corrects random fluctuations by incorporating the influence of neighboring area values, thus providing the estimates with greater stability and robustness. A spatial proximity matrix based on the contiguity criterion was then used, assigning unity (value 1) to first-order neighboring municipalities, i.e., contiguous municipalities, and assigning a null value (0) to the others. The matrix was built according to the "queen" contiguity convention, which considers as neighbors all municipalities that share any point of contact, whether by line or vertex, thus providing greater sensitivity to the spatial configuration of the data.

The same spatial weighting matrix was later used as the basis for calculating the global and local Moran's spatial autocorrelation indices, as well as the Getis-Ord Gi technique. These approaches are widely used in spatial epidemiology studies to detect patterns of event clustering, which helps in identifying areas with higher or lower risk concentrations.

The global Moran's I index assesses the existence of spatial autocorrelation in the dataset, identifying whether there is a significant spatial pattern. This indicator ranges from -1 to +1, where: values ​​close to +1 suggest strong positive spatial autocorrelation (direct patterns), values ​​close to 0 indicate absence of autocorrelation, and values ​​close to -1 reflect strong negative spatial autocorrelation (indirect patterns). This interpretation is important for understanding the terms "weak", "moderate" and "strong" used in the results. When global autocorrelation was detected, the local Moran's I index (LISA - Local Indicators of Spatial Association) was used to detail the intensity of spatial association at the municipal level. This approach allows identification of local patterns such as: high-high and low-low, which indicate clusters of municipalities with similar values ​​(positive autocorrelation); and high-low and low-high, which reveal spatially discrepant points (negative autocorrelation), that is, municipalities the values of which ​​differ significantly from the values ​​of their neighbors ^11^.

In turn, the Getis-Ord Gi technique calculates z-scores for each spatial unit, which are used to identify hot and cold areas. High z-scores indicate that a given location and its neighbors have significantly high rates and form high-risk clusters, while negative z-scores highlight areas where low rates predominate, in the municipality and its surroundings. This analysis is particularly useful for guiding public health surveillance and intervention actions, as it highlights spatial concentrations of risk that would not be easily detected by traditional methods ^12^.

GeoDa software was used to perform spatial autocorrelation analyses. QGIS was used for processing, organizing and visualizing the spatial data. Environment and thematic maps and cartographic representations of the clusters identified were created in this environment. The natural breaks method was adopted for classifying the intervals on the maps. It optimizes the distribution of values ​​by minimizing variance within each class and maximizing variance between classes, allowing for a more accurate representation of the spatial differences observed.

## Results 

A total of 7,088,623 deaths were recorded in Brazil between 2003 and 2023 (Table 1). 

Predominance of deaths was observed among males (52.5%), accounting for a standardized mortality rate of 181 deaths/100,000 inhabitants, compared to females (157 deaths/100,000). Mortality increased progressively with age. Rates were estimated at values ​​below eight deaths per 100,000 inhabitants up to age 19. From age 40 onwards, rates increased sharply, with 199 deaths/100,000 in the 40-49 age group, and 1,243 deaths/100,000 inhabitants in the 60-69 age group. The highest rate was observed in the 70-79 age group (3,420.2/100,000 inhabitants). An unexpected reduction in the mortality rate was also noted in the over 80 group (4.1/100,000 inhabitants) (Table 1).

Most deaths occurred among people of White (53.6%) and mixed-race (32.4%) race/skin color. The highest mortality rates were observed among those of Black (200/100,000 inhabitants) and White skin color (199/100,000 inhabitants). The highest proportion of deaths was also recorded among people with 1 to 3 years of schooling (23.4%) and among those with unknown schooling levels (23.3%). An inverse relationship was observed between schooling and mortality: a rate of 415/100,000 inhabitants was found among individuals with 1 to 3 years of schooling, while those with 12 years or more had the lowest rate (21.2/100,000 inhabitants) (Table 1).

The average CVD mortality rate in Brazil, between 2000 and 2023, was equivalent to 169 deaths per 100,000 inhabitants, with an annual average of 337,553 deaths. The highest proportion of deaths occurred in the Southeast region, which accounted for 47.4% of the records, followed by the Northeast (25.4%) and Southern (15.8%) regions. Standardized mortality rates varied considerably between regions. The highest rates were recorded in the Southeast (190 per 100,000 inhabitants) and the South (187 per 100,000 inhabitants). The lowest rate was observed in the Northern region, with 96 deaths per 100,000 inhabitants (Table 2).

Relative stability was observed in CVD mortality rates in Brazil and by region over time, with subtle variations between years. The graph also emphasized the persistence of high rates in the South and Southeast regions. The Northeast region showed a moderate rising trend in rates between 2003 and 2015, followed by stabilization. The Midwest region had a similar pattern, but with slightly lower rates. The Northern region presented the lowest CVD mortality rates throughout the entire period analyzed (Figure 1).

**Table 1:** Cardiovascular disease sociodemographic characterization and standardized mortality rate per 100,000 inhabitants. Brazil, 2003-2023 (n=7,088,623)

Variable	n (%)	Mortality rate
Sex		
Male	3,721,949 (52.5)	181.9.
Female	3,365,931 (47.4)	157.4
Unknown	743 (0.1)	
Age group (years)		
<4	3,322 (0.1)	1.0
5-9	5,789 (0.1)	1.6
10-14	13,262 (0.1)	3.6
15-19	53,819 (0.7)	7.5
20-29	147,449 (2.0)	22.0
30-39	412,471 (5.8)	75.5
40-49	854,729 (12.0)	199.0
50-59	1,375,999 (19.4)	510.7
60-69	1,819,388 (25.6)	1,243.2
70-79	2,383,324 (33.6)	3,420.2
>80	6,374 (0.1)	4.1
Unknown	12,697 (0.1)	1.0
Race/skin color		
White	3,801,491 (53.6)	199.7
Black	605,667 (8.5)	200.9
Asian	42,541 (0.6)	96.2
Mixed-race	2,299,138 (32.4)	132.1
Indigenous	12,290 (0.1)	71.2
Unknown	327,496 (4.6)	
Schooling (years)		
None	1,360,522 (19.1)	137.8
1-3	1,659,502 (23.4)	415.9
4-7	1,348,108 (19.0)	168.9
8-11	767,902 (10.8)	85.0
12 or more	299,363 (4.2)	21.2
Unknown	1,653,226 (23.3)	

**Table 2:** Number of deaths, mean deaths and standardized mortality rate per 100,000 inhabitants due to cardiovascular diseases, by region. Brazil, 2003-2023 (n=7,088,623)

Region	n (%)	Mean deaths	Mortality rate
North	342,127 (4.8)	16,292	96.9
Northeast	1,807,161 (25.4)	86,055	157.6
Southeast	3,365,596 (47.4)	160,266	190.1
South	1,124,748 (15.8)	53,559	187.5
Midwest	448,991 (6.3)	21,381	142.6
Brazil	7,088,623 (100.0)	337,553	169.4

**Figure 1:**
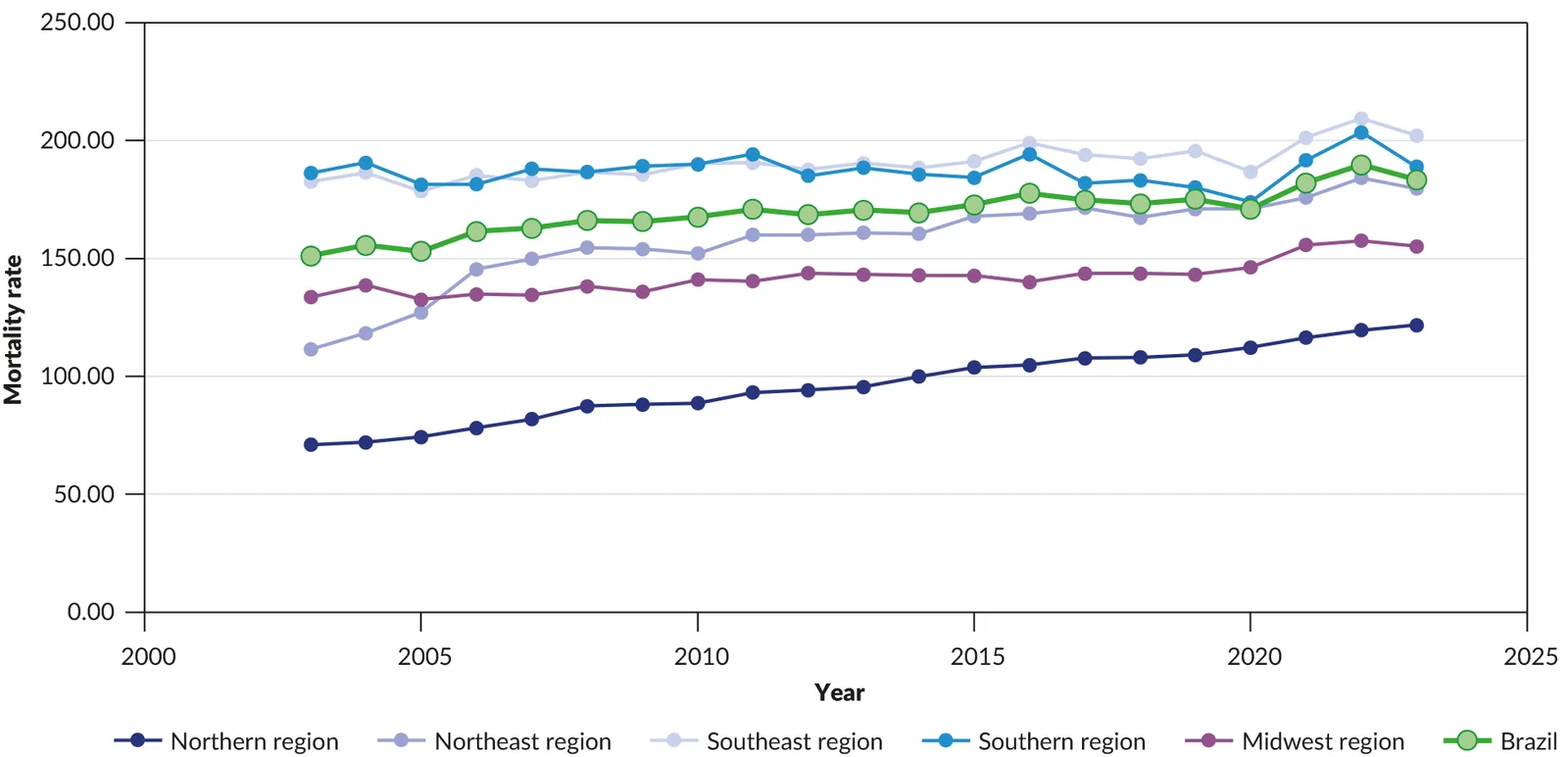
Cardiovascular disease mortality trend by region. Brazil, 2003-2023 (n=7,088,623)

Nationally, the joinpoint analysis identified two distinct trend segments. Between 2003 and 2008, annual growth of 1.97% was observed (95% CI 1.19; 3.77). This increase was followed by a period of stagnation, until, between 2020 and 2023, a new increase of 2.34% per year occurred (95%CI 0.97; 4.34). In the North and Northeast regions, a similar pattern was observed, with growth concentrated in the time series. In the North, the rate increased by 4.38% per year (95%CI 3.32; 7.02) between 2003 and 2008, while in the Northeast the increase was 8.15% per year (95%CI 6.24; 10.69) from 2003 to 2007. After these initial periods, the trend became stationary in both regions. In the other regions, growth occurred in the most recent period. In the Southeast, an annual increase of 2.16% (95%CI 0.57; 4.32) was recorded between 2020 and 2023. In the South, the increase was 2.95% per year (95%CI 1.21; 6.76) between 2019 and 2023. In the Midwest, an annual increase of 2.38% (95%CI 0.89; 4.93) was observed in the period from 2019 to 2023 (Table 3).

In the spatial analysis of the event, a global Moran's I index of 0.02 (p-value< 0.05) was obtained, indicating weak, but positive, spatial autocorrelation. This value suggested that, although there was some tendency for municipalities with similar rates to cluster together, this association was not very significant on a national scale. This result was consistent with the heterogeneous and widespread nature of the event, since CVDs are the leading cause of death in Brazil and affect different geographic, population and socioeconomic contexts.

This interpretation was highlighted by comparing the maps of unadjusted and smoothed mortality rates. The map of the unadjusted rates (Figure 2A) showed high variability between municipalities, with an apparent concentration of high values ​​in the Midwest, especially in Mato Grosso and Goiás states. This unadjusted distribution, however, is sensitive to random fluctuations, particularly in localities with small populations, which may overestimate or underestimate the real risk. The map with smoothed rates (Figure 2B), on the other hand, presented a more homogeneous and epidemiologically consistent spatial configuration, revealing regional clusters more clearly. Even after smoothing, areas with high mortality persisted in the two aforementioned states, emphasizing that they correspond to zones of concentrated CVD mortality burden.

**Table 3:** Annual percentage change (APC) and 95% confidence intervals (95%CI) of mortality due to cardiovascular diseases, by region. Brazil, 2003-2023 (n=7,088,623)

Region	Period	APC (95%CI)	p-value	Trend
North	2003-2008 2008-2023	4.38 (3.32; 7.02) 2.33 (2.03; 2.54)	<0.001 0.007	Rising Rising
Northeast	2003-2007 2007-2023	8.15 (6.24; 10.69) 1.11 (0.92; 1.25)	<0.001 <0.001	Rising Rising
Southeast	2003-2020 2020-2023	0.40 (0.08; 0.55) 2.16 (0.57; 4.32)	0.065 0.004	Stationary Rising
South	2003-2016 2016-2019 2019-2023	0.12 (-0.13; 2.65) -2.06 (-3.42; -0.17) 2.95 (1.21; 6.76)	0.216 0.024 0.008	Stationary Falling Rising
Midwest	2003-2019 2019-2023	0.47 (-0.11; 0.67) 2.38 (0.89; 4.93)	0.077 0.001	Stationary Rising
Brazil	2003-2008 2008-2020 2020-2023	1.97 (1.19; 3.77) 0.48 (-0.57; 0.67) 2.34 (0.97; 4.34)	0.003 0.157 <0.001	Rising Stationary Rising

**Figure 2:**
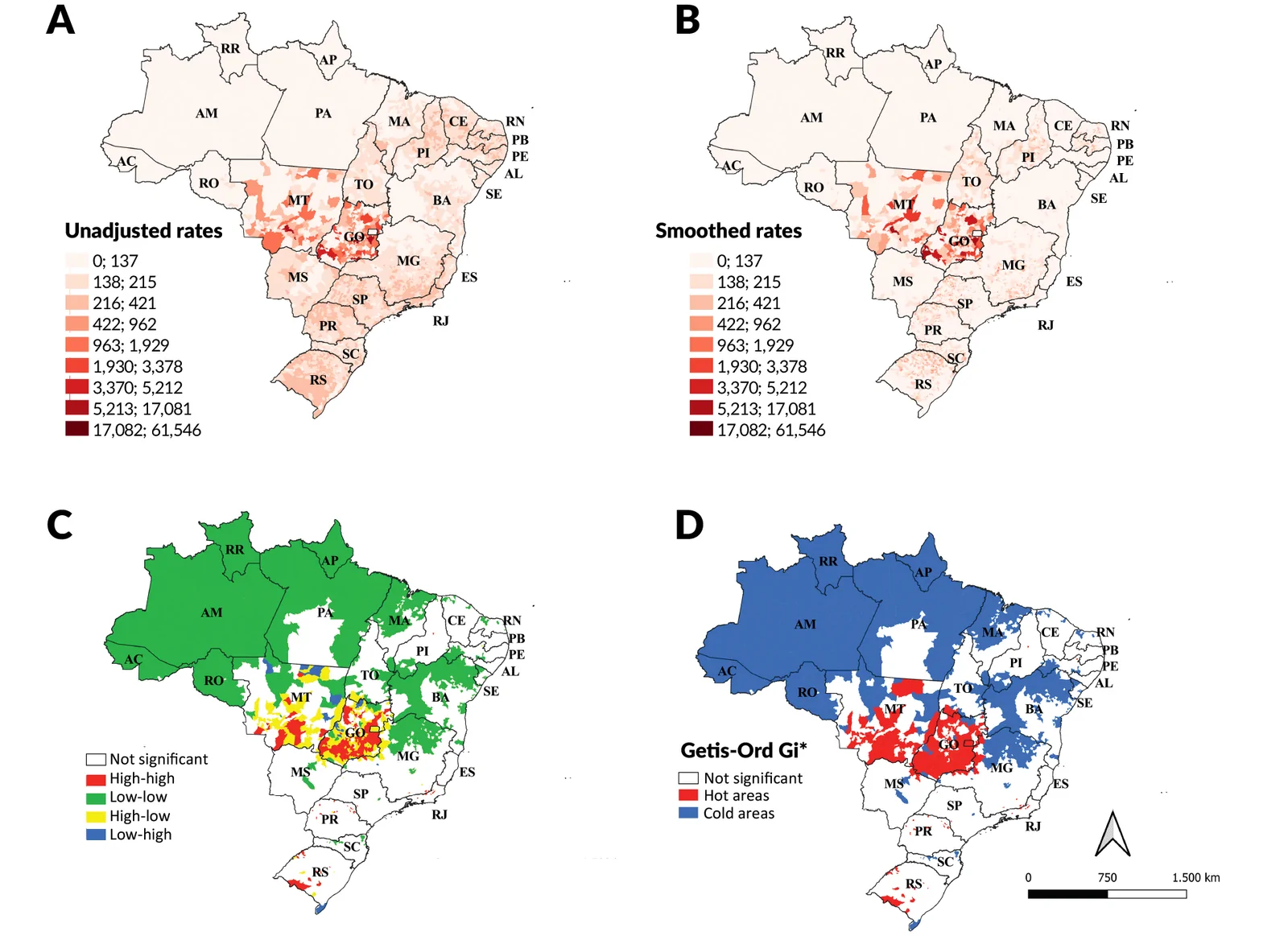
Distribution of unadjusted rates (A) and smoothed rates (B) and identification of spatial clusters of high and low risk of mortality due to cardiovascular diseases according to Local Moran's I (C) and Getis-Ord Gi. Brazil, 2003-2023 (n=7,088,623)

Local spatial autocorrelation analysis enabled identification of geographic patterns in CVD mortality in Brazil. The first map (Figure 2C) illustrated statistically significant clusters of municipalities with high rates, surrounded by municipalities with equally high rates. These clusters are mainly concentrated in southern Mato Grosso and in Goiás. Low-low clusters were concentrated mainly in the Northern region of Brazil and in some Northeastern states, such as Maranhão and Bahia.

Patterns of transition or spatial dissonance were also identified, such as high-low clusters in Mato Grosso and Goiás states, and low-high clusters in Mato Grosso and Rio Grande do Sul states, representing areas where municipalities with high (or low) mortality are surrounded by neighbors with opposite patterns (Figure 2D). The map summarized these findings using a binary approach based on local significance (Getis-Ord Gi) and classified the municipalities into hot and cold areas. The hot areas were concentrated in the same regions previously highlighted as high-high, with high density in the Midwest region. The cold areas, which indicated statistically significant clusters of low mortality, were widely distributed in the Northern region, on the Northeast coast and in the Southern region of the country.

## Discussion 

The findings of this study show that CVD mortality in Brazil remains high and heterogeneously distributed between population groups and territories. A greater concentration of the outcome was observed in socially vulnerable segments, reflecting structural inequalities that cut across sex, age and education. Regionally, marked contrasts persist, with relative stabilization in historically more affected areas and progressive increase in previously less impacted regions, indicating a redistribution of the disease burden in the country. Spatial analysis revealed geographic patterns that highlighted higher-risk areas and areas of lower mortality, suggesting that socioeconomic, structural and health service organization factors interact differently in different territories. These results, taken together, highlighted that cardiovascular disease mortality in Brazil is influenced by social and contextual determinants and that its current distribution expresses a complex scenario of epidemiological transition and persistent inequalities.

Among the limitations of this study, the possibility of underreporting or inadequate classification of underlying causes of death stands out, especially in municipalities with lower diagnostic capacity and service coverage. It is also important to consider that Mortality Information System (SIM) coverage presents historically documented regional variations, which includes differences in the under-recording of deaths, unequal proportion of ill-defined causes, as well as heterogeneity in the completeness of death certificate fields. These variations may influence the spatial distribution of CVD mortality found, which should be interpreted as part of the context that shapes the findings. Because aggregated data were used, it was not possible to control for individual or contextual factors that could explain local differences, which implied a risk of ecological fallacy. Future studies could delve deeper into territorial factors associated with the clusters identified and explore trends in specific population subgroups.

In this study, higher risk of CVD mortality was observed among males, a finding that is consistent with results from national authors ^13^, who identified greater male vulnerability to CVD based on an epidemiological study using aggregated mortality data. This difference can be understood by the interaction between biological factors, such as lower hormonal protection before menopause in women, and behavioral factors, such as higher prevalence of smoking, alcohol abuse and inadequate diet among men, as well as lower adherence to preventive practices and health monitoring, which increases cardiovascular risk over lifetime ^14^.

The analysis of the data in this research showed an increase in CVD mortality with advancing age, this being a pattern consistent with those widely described in the literature ^15^. Aging intensifies accumulated exposure to conditions such as hypertension, diabetes and dyslipidemia, in addition to leading to a progressive reduction in cardiovascular functional reserve and an increase in the comorbidity burden, these being elements that combine to explain the greater vulnerability of the elderly to these outcomes. Interestingly, this study found a lower mortality rate in the over 80 age group, an unexpected finding that deserves attention. This result may be related to underreporting or inadequate classification of underlying causes at advanced ages, as well as the possible "survivor effect", whereby individuals who have achieved greater longevity tend to have a more robust profile and a lower burden of risk factors. These aspects may explain the discrepancy observed and emphasize the need for caution when interpreting rates in elderly age groups ^16^^-^^17^.

The results identified higher CVD mortality among people of Black race/skin color, emphasizing the influence of social determinants on CVD distribution. This pattern is consistent with an American cohort study of 50,808 people, which indicated poorer socioeconomic conditions, less access to health services and greater exposure to environments and practices that also increase cardiovascular risk among Black populations in Brazil ^16^. It is important to recognize that classification by race/skin color is based on self-reporting and may not fully capture experiences of discrimination, indicating the need for more in-depth intersectional analyses.

The findings of this study showed an inverse relationship between education and cardiovascular mortality, a pattern that follows the trend indicated by the international literature, which recognizes education as a central marker of health inequalities in population studies carried out between 2003 and 2019 ^18^. Lower schooling tends to be associated with less access to information, resources, services and healthy environments, which explains the higher mortality observed among people with up to three years of schooling and stresses the importance of educational and health promotion strategies sensitive to the population's education level ^19^.

In Brazil, the regional patterns of CVD mortality identified in this study, especially the growth in historically less affected regions, showed similarities with middle-income countries in Asia and demonstrated that CVD mortality tends to be higher in contexts with fragmented health systems, low primary health care coverage and a greater concentration of adverse social determinants, these being characteristics that are also reflected in part of the Brazilian scenario ^20^. In contrast to the findings of this study, which showed an increase in CVD mortality in certain Brazilian regions, middle- and high-income countries that have advanced in public policies on prevention and tobacco control, increased access to essential cardiovascular medications and strengthened primary health care have demonstrated sustained reductions in CVD mortality in recent decades ^21^^-^^22^.

In this study, temporal analysis revealed stabilization or a slight reduction in CVD mortality rates in Southeast and Souternh Brazil, in contrast to the continuous growth observed in the North and Northeast throughout the period analyzed. This divergence may be related to structural differences and access to health services, as well as the advancement of prevention policies and risk factor management in the more developed regions of the country ^4^^,^^18^. The increase in mortality rates in less favored regions may also reflect behavioral and environmental changes, in addition to persistent inequalities in access to timely care.

The increase in cardiovascular mortality observed in the North and Northeast regions may be influenced by accelerated demographic transformations, heterogeneous urbanization and nutritional transition, added to the progressive improvement of health information systems in these territories. Nevertheless although improvements in recording may increase sensitivity for identifying underlying cardiovascular causes, the increasing pattern is consistent with a real expansion of the disease burden ^23^^-^^24^.

In the results of this study, spatial analysis revealed a heterogeneous territorial distribution of CVD mortality, with clusters of higher mortality especially in municipalities of Mato Grosso and Goiás states. The spatial pattern identified does not occur in isolation: similar findings were described in a national ecological study that analyzed cerebrovascular disease mortality in Brazil between 1996-2015 and identified higher risk in areas with poorer human development and greater social vulnerability ^25^. Similarly, an observational study with aggregated data from the first year of the COVID-19 pandemic pointed to marked regional inequalities in CVD mortality, associated with healthcare capacity and structural disparities in local health systems ^26^. Together, these findings highlighted that socioeconomic inequalities, irregular healthcare coverage and differences in health infrastructure configure micro-territories of higher cardiovascular risk, which influenced the distribution of deaths.

Goiás and Mato Grosso states have experienced intense socio-spatial transformations in recent decades, driven by agribusiness, growth of medium-sized cities and significant migratory flows. This process of economic expansion generates a rapid increase in urbanization and, commonly, changes in the local way of life, with greater consumption of ultra-processed foods, reduced physical activity and an increase in cardiometabolic risk factors ^27^. These profiles have been observed, above all, in municipalities with a strong presence of the agro-industrial sector, where higher average income does not necessarily translate into a proportional improvement in health indicators ^28^.

In addition, Goiás and Mato Grosso states show significant internal heterogeneity. In Goiás, there are striking contrasts between the Goiânia health macro-region, with better healthcare infrastructure, and regions such as northern and northeastern Goiás, where healthcare gaps persist, along with long distances to specialized services and a lower density of healthcare professionals. In Mato Grosso, the extensive territorial size and population dispersion exacerbate challenges in health care organization: many municipalities depend on regional centers such as Cuiabá, Sinop and Rondonópolis, which generates significant delays in the care pathway ^29^^-^^31^.

These states also have heterogeneous primary health care coverage. In rural and peri-urban areas, weaknesses in the Family Health Strategy, staff turnover, difficulty in continuity of care, and low capacity to manage chronic conditions, especially hypertension and diabetes, which are direct precursors of cardiovascular complications, are frequent ^32^.

Furthermore, persistent social inequalities are compounded, with pockets of poverty in areas of agricultural expansion, traditional communities and rural settlements, where access to health services is more limited. This set of factors - accelerated epidemiological transition, changes in lifestyles, territorial heterogeneities in access to health, dependence on regional centers, and fragility of primary health care - offers plausible hypotheses to explain the higher concentration of CVD mortality in the municipalities of Mato Grosso and Goiás states, as evidenced in the national ecological study carried out in 2022 with DATASUS data, which related diseases of the circulatory system and social determinants focusing on vulnerability ^33^.

Given the regional inequalities identified, expanding and improving primary health care across the territory is a key strategy, with improved diagnostic capacity and implementation of care pathways for hypertension, diabetes and atherosclerotic disease. In parallel, access to specialized services needs to be improved through regionalization of cardiovascular care, as well as effective integration between levels of care, in order to ensure continuity of care. Structural measures, such as active surveillance of risk factors, evidence-based health promotion programs, and reduction of social inequalities that shape cardiovascular risk, are also fundamental to reducing interstate and intraregional differences. These paths point to the need for coordinated policies that articulate prevention, longitudinal care and equity in the provision of services ^34^.

This study demonstrated that cardiovascular disease (CVD) mortality in Brazil remains high and is concentrated mainly among men, the elderly and individuals with low schooling levels. A rising trend was observed in the North, Northeast and Midwest regions, in addition to spatial clusters of high mortality in municipalities of Mato Grosso and Goiás states. These results highlighted sociodemographic and territorial inequalities that need to guide health surveillance and planning actions.

## Data Availability

The databases used for analysis area are available at the SciELO Data repository: https://doi.org/10.48331/SCIELODATA.MKZUPS.
