# Supplementary material for: Cardiovascular disease mortality: time series and spatial analysis, Brazil, 2003-2023
Source: Epidemiol Serv Saude. 2026 Mar 15;35:e20250892. doi: 10.1590/S2237-96222026v35e20250892.en (PMC13686314; doi:10.1590/S2237-96222026v35e20250892.en)
Supplement: Translation

# Translation: Mortalidade por doenças cardiovasculares: análise de série temporal e espacial, Brasil, 2003-2023

**DOI:** 10.1590/S2237-96222026v35e20250892.pt

**Authors:** Francisca Vaneska Lima Nascimento, Ítalo Rigoberto Cavalcante Andrade, Lara Lídia Ventura Damasceno, Thiago Santos Garces, Thereza Maria Magalhães Moreira, George Jó Bezerra Sousa, Maria Lúcia Duarte Pereira, Lúcia de Fátima da Silva

**Affiliations:** 1 Universidade Estadual do Ceará, Programa de Pós-Graduação Cuidados Clínicos em Enfermagem e Saúde, Fortaleza, CE, Brasil Universidade Estadual do Ceará Programa de Pós-Graduação Cuidados Clínicos em Enfermagem e Saúde Fortaleza CE Brasil; 2 Ministério da Saúde, Brasília, DF, Brasil Ministério da Saúde Brasília DF Brasil

**Keywords:** Doenças Cardiovasculares, Mortalidade, Análise Espacial, Fatores de Tempo, Análise de Dados Secundários.

## Abstract

**Objetivo:**

Descrever a distribuição temporal e espacial da mortalidade por doenças cardiovasculares no Brasil de 2003 a 2023.

**Métodos:**

Estudo de análise de série temporal e espacial, conduzido a partir de dados secundários do Sistema de Informações sobre Mortalidade do Departamento de Informática do Sistema Único de Saúde, com inclusão de todos os óbitos por doenças do aparelho circulatório. Foram analisadas características sociodemográficas e calculadas taxas de mortalidade padronizadas por 100 mil habitantes. A tendência temporal foi avaliada por meio de regressão por pontos de inflexão, com estimativa da variação percentual anual. A análise espacial considerou taxas médias municipais, brutas e suavizadas pelo método bayesiano empírico local, além da autocorrelação espacial global e local.

**Resultados:**

Foram registrados 7.088.623 óbitos por doenças cardiovasculares no Brasil entre 2003 e 2023, com predominância no sexo masculino e de maior mortalidade em idosos e pessoas com baixa escolaridade. As maiores taxas foram observadas nas regiões Sudeste (190,14/100 mil) e Sul (187,59/100 mil), enquanto as regiões Norte (APC 2003-2008 4,38; APC 2008-2023 2,33) e Nordeste (APC 2003-2007 8,15; APC 2007-2023 1,11) apresentaram tendência crescente ao longo do período (p-valor<0,001). O índice de Moran global (0,02; p-valor<0,05) apontou fraca autocorrelação espacial positiva, mas estatisticamente significativa. A análise local revelou agrupamentos de alta mortalidade concentrados principalmente em municípios de Mato Grosso e de Goiás, enquanto áreas de baixa mortalidade predominaram no Norte e no litoral do Nordeste.

**Conclusão:**

A mortalidade por doenças cardiovasculares no Brasil apresentou distribuição heterogênea no tempo e no espaço.

**Ethical aspects**

Esta pesquisa utilizou bancos de dados de domínio público e anonimizados.

## Introdução 

As doenças cardiovasculares (DCV) abrangem um conjunto de agravos que comprometem o coração e os vasos sanguíneos, caracterizado por alterações estruturais ou funcionais do sistema circulatório. Esse grupo engloba a cardiopatia isquêmica, a insuficiência cardíaca, as doenças hipertensivas, as doenças cerebrovasculares, as doenças vasculares periféricas, dentre outras. As DCV constituem a principal causa de mortalidade global. e sua ocorrência é fortemente influenciada por fatores de risco modificáveis, como hipertensão arterial, diabetes mellitus, dislipidemias, tabagismo, inatividade física, obesidade e alimentação inadequada, além de determinantes sociais e ambientais, que incluem condições socioeconômicas, escolaridade, raça/cor da pele, acesso aos serviços de saúde e desigualdades territoriais ^1^.

Em 2019, as DCV foram responsáveis por cerca de 17 milhões de óbitos, 32% das mortes mundiais, das quais mais de 80% ocorreram em países de baixa e de média renda e quase um terço foi considerado morte prematura (≤70 anos) ^2^. Em 2021, esse número avançou para 20 milhões de mortes, um aumento acumulado de quase 60% em relação aos níveis observados desde 1990 ^3^. No Brasil, o panorama acompanha essa tendência. Estimativas indicam que, em 2021, a taxa padronizada de mortalidade por doenças coronárias foi de 67/100 mil habitantes e que as causas cardiovasculares representaram 28% dos óbitos por doenças não transmissíveis, o que contribuiu significativamente para a mortalidade evitável e para os anos potenciais de vida perdidos ^4^.

Em termos econômicos e de impacto em saúde pública, estudos de diferentes períodos ajudam a compor esse cenário crescente. Em 2015, o custo das DCV foi estimado em R$ 37 bilhões, dos quais 61% referiam-se a mortes prematuras e 39% correspondiam a despesas diretas e de perda de produtividade ^4^. Em outra estimativa nacional, a elevada ingestão de sódio resultou, em 2017, em 46.651 mortes evitáveis e em 575.172 perdas ajustadas por anos de vida perdidos ^5^. Projeções sugerem que, entre 2025 e 2050, a mortalidade cardiovascular poderá aumentar em até 73%, o que significaria 35 milhões de óbitos, enquanto as perdas ajustadas por anos de vida podem crescer em 54% ^3^.

Com a consideração da relevância epidemiológica e econômica das DCV, torna-se fundamental identificar como os indicadores de mortalidade se distribuem entre diferentes estratos populacionais, especialmente frente ao envelhecimento acelerado, às desigualdades regionais, às perdas de anos potenciais de vida e aos custos diretos e indiretos associados. Essa compreensão é essencial para subsidiar a tomada de decisão em saúde pública e orientar políticas mais eficazes e ajustadas às especificidades territoriais. A utilização de dados secundários permite captar de forma contínua e abrangente as dinâmicas da mortalidade cardiovascular, o que oferece suporte para ações de prevenção, de detecção precoce, de manejo clínico e para adequada alocação de recursos.

Esse delineamento permite a avaliação integrada da distribuição da mortalidade cardiovascular no Brasil, o que possibilita a identificação de regiões e de populações mais vulneráveis. Tal abordagem tem sido uma prioridade desde a adoção do Plano de Ação Global para o Controle das Doenças Crônicas Não Transmissíveis (DCNT) 2013-2020 pela Organização Mundial da Saúde ^6^. Em continuidade, a Agenda 2030 para o Desenvolvimento Sustentável ampliou essa diretriz, ao propor a redução de um terço da mortalidade prematura por essas doenças até 2030. No Brasil, esse compromisso internacional foi incorporado por meio do Plano de Ações Estratégicas para o Enfrentamento das DCNT no Brasil 2021-2030 ^7^.

Este estudo objetivou descrever a distribuição temporal e espacial da mortalidade por DCV no Brasil. 

## Métodos 

Delineamento do estudo

Estudo de análise de série temporal e espacial, conduzido a partir de dados secundários provenientes do Sistema de Informações sobre Mortalidade (SIM), disponibilizados pelo Departamento de Informática do Sistema Único de Saúde (DATASUS), acessados via plataforma Tabnet. 

O SIM é o sistema oficial brasileiro de vigilância de óbitos, criado para monitorar o perfil de mortalidade no país. Seu funcionamento baseia-se na declaração de óbito, documento padronizado em nível nacional e emitido por profissionais de saúde. Após o preenchimento, a declaração de óbito é encaminhada às secretarias municipais de saúde, onde passa por etapas de codificação das causas básicas e dos contribuintes, de acordo com a 10ª edição da Classificação Internacional de Doenças (CID-10). Os dados são depois consolidados pelas secretarias estaduais e pelo Ministério da Saúde, que realiza rotinas de crítica, de validação e de padronização para disponibilização pública ^8^.

Contexto

Como espaço geográfico de interesse, foram consideradas as cinco macrorregiões do Brasil (Norte, Norte, Sul, Sudeste e Centro-Oeste) para a série temporal, e os 5.570 municípios brasileiros para a análise espacial. 

Fonte de dados

Foram considerados todos os óbitos por doenças do aparelho circulatório, presentes no capítulo IX da CID-10, registrados entre os anos de 2003 e 2023. Esse capítulo integra a seguinte lista de morbidades da CID-10: doença reumática crônica do coração (I05-I09); hipertensão essencial (I10); outras doenças hipertensivas (I11-I15); infarto agudo do miocárdio (I21); outras doenças isquêmicas do coração (I20, I22-I25); embolia pulmonar (I26); transtornos de condução e arritmias cardíacas (I44-I49); insuficiência cardíaca (I50); outras doenças do coração (130-I43, I51); outras doenças do aparelho circulatório (I70-I99) ^9^. Os bancos de dados utilizados para construção dos resultados estão disponíveis no repositório SciELO Data e são de acesso público (https://doi.org/10.48331/SCIELODATA.MKZUPS) ^9^.

Métodos estatísticos

Para caracterização sociodemográfica, utilizaram-se as variáveis: sexo (masculino; feminino), faixa etária (<4 anos; 5-9 anos; 10-14 anos; 15-19 anos; 20-29 anos; 30-39 anos; 40-49 anos; 50-59 anos; 60-69 anos; 70-79 anos; >80 anos), raça/cor da pele (branca; preta; parda; amarela; indígena) e escolaridade (nenhuma; 1-3 anos; 4-7 anos; 8-11 anos; ≥12). As informações demográficas para subsidiar o cálculo das taxas de mortalidade foram obtidas no portal DATASUS, na seção "Demográficas e Socioeconômicas", que dispõe sobre a projeção da população por sexo, idade simples ou faixa-etária de 2000 a 2070. 

As taxas de mortalidade foram padronizadas para cada variável analisada, o que permitiu comparações mais precisas entre os diferentes grupos sociodemográficos, e foram calculadas com inserção, no numerador, do número de óbitos em cada estrato sociodemográfico e, no denominador, das respectivas populações pertencentes às mesmas categorias demográficas, conforme disponibilizado pelas projeções populacionais.

Para as análises de tendência temporal realizadas no Programa de Regressão por Pontos de Junção (*Joinpoint Regression Program*), foi analisado o período completo de 2003 a 2023, com testagem de modelos com zero até o número máximo permitido de pontos de inflexão. Utilizou-se o método tradicional baseado em permutações de Monte Carlo, que permite a obtenção dos p-valores associados à variação percentual anual (APC). As APC apresentadas neste estudo foram obtidas com base no teste de permutação, acompanhadas de seus respectivos intervalos de confiança de 95% (IC95%) e seus p-valores. 

Foi considerado no ajuste dos modelos o componente autorregressivo de primeira ordem (AR=1), a fim de minimizar possíveis efeitos de autocorrelação serial,. Esse procedimento busca corrigir dependências temporais entre observações consecutivas, o que aumenta a precisão das estimativas. Já as taxas de mortalidade foram transformadas pelo logaritmo natural (ln), conforme procedimento padrão do modelo log-linear do software. Essa transformação permite interpretar as inclinações dos segmentos, bem como realizar comparações diretas entre diferentes magnitudes de mudança ao longo do tempo. Para tanto, definiu-se como variável independente o ano de notificação, e, como dependente, a taxa de mortalidade.

Utilizou-se o ano de notificação do óbito, conforme disponibilizado no SIM/Tabnet, que representa a informação consolidada pelo Ministério da Saúde após o fluxo de processamento das declarações de óbito. Embora o ano de ocorrência seja conceitualmente distinto, estudos baseados em dados anuais agregados do SIM demonstraram mínima discrepância entre ambas as datas, especialmente em bases já consolidadas, o que não compromete a análise temporal.

A direção da tendência dependeu do valor de APC e da significância estatística. Quando os valores são positivos e estatisticamente significantes (p-valor<0,05), indica-se uma trajetória de crescimento. Se forem negativos e estatisticamente significantes, apontam para um declínio ao longo do tempo. Caso a significância não seja constatada (p-valor>0,05), considera-se que não há alteração relevante no período analisado, o que caracteriza uma tendência estável ou estacionária ^10^. 

Na análise espacial, foi calculada a taxa de mortalidade média anual ajustada pela população residente no ano central para cada município, a fim de considerar as diferenças nas características demográficas das populações e reduzir o viés das disparidades, conforme a fórmula: média de óbitos por município (total de óbitos/número de anos) dividida pela população residente no ano central, multiplicado pelo coeficiente de 100 mil habitantes.

Com o objetivo de reduzir a instabilidade inerente às taxas brutas, aplicou-se a suavização das taxas por meio do método bayesiano empírico local. Esse procedimento corrige flutuações aleatórias ao incorporar a influência dos valores das áreas vizinhas, o que confere maior estabilidade e robustez às estimativas. Utilizou-se, então, uma matriz de proximidade espacial baseada no critério de contiguidade, em que foi atribuída a unidade (valor 1) aos municípios vizinhos de primeira ordem, ou seja, aqueles que compartilham uma fronteira direta, e valor nulo (0) aos demais. A matriz foi construída segundo a convenção "rainha", que considera como vizinhos todos os municípios que compartilham qualquer ponto de contato, seja por linha ou vértice, o que promove maior sensibilidade à configuração espacial dos dados.

Essa mesma matriz de pesos espaciais foi posteriormente utilizada como base para os cálculos dos índices de autocorrelação espacial de Moran global e local, bem como da técnica Getis-Ord Gi. Essas abordagens são amplamente empregadas em estudos de epidemiologia espacial para detectar padrões de aglomeração de eventos, o que auxilia na identificação de áreas com maior ou menor concentração de risco.

O índice de Moran global avalia a existência de autocorrelação espacial no conjunto dos dados, identifica se há um padrão espacial significativo. Esse indicador varia de -1 a +1, em que: valores próximos de +1 sugerem forte autocorrelação espacial positiva (padrões diretos), valores próximos de 0 indicam ausência de autocorrelação e valores próximos de -1 refletem forte autocorrelação espacial negativa (padrões indiretos). Essa interpretação é importante para compreender os termos "fraco", "moderado" e "forte" utilizados nos resultados. Quando detectada autocorrelação global, empregou-se o índice de Moran local (LISA - *Local Indicators of Spatial Association*) para detalhar a intensidade da associação espacial em nível municipal. Essa abordagem permite identificar padrões locais como: alto-alto e baixo-baixo, que indicam agrupamentos de municípios com valores semelhantes (autocorrelação positiva); e alto-baixo e baixo-alto, que revelam pontos espaciais discrepantes (autocorrelação negativa), ou seja, municípios cujos valores diferem significativamente dos valores de seus vizinhos ^11^.

Já a técnica Getis-Ord Gi calcula escores z para cada unidade espacial, os quais são utilizados para a identificação de áreas quentes e de áreas frias. Valores elevados de z indicam que determinada localidade e suas vizinhas possuem taxas significativamente altas e formam agrupamentos de risco elevado, e escores z negativos destacam áreas onde taxas baixas predominam, no município e no seu entorno. Essa análise é particularmente útil para orientar ações de vigilância e de intervenção em saúde pública, uma vez que evidencia concentrações espaciais de risco que não seriam facilmente detectadas por métodos tradicionais ^12^.

O software GeoDa foi empregado para a realização de análises de autocorrelação espacial. O QGIS foi utilizado para o processamento, a organização e a visualização dos dados espaciais. Nesse ambiente, foram elaborados os mapas temáticos e as representações cartográficas dos aglomerados identificados. Para a classificação dos intervalos nos mapas, adotou-se o método de quebras naturais, que otimiza a distribuição dos valores ao minimizar a variância dentro de cada classe e maximizar a variância entre classes, o que permite uma representação mais fiel das diferenças espaciais observadas.

## Resultados 

Foram registrados 7.088.623 óbitos no Brasil entre 2003 e 2023 (Tabela 1). 

Observou-se predominância de óbitos entre indivíduos do sexo masculino (52,5%), responsáveis por taxa de mortalidade padronizada de 181 óbitos/100 mil habitantes, em comparação às mulheres (157 óbitos/100 mil). A mortalidade aumentou progressivamente com a idade. As taxas foram estimadas em valores inferiores a oito óbitos por 100 mil habitantes até os 19 anos. A partir dos 40 anos, as taxas elevaram-se de forma acentuada, 199 óbitos/100 mil entre 40 e 49 anos, e 1.243 óbitos/100 mil habitantes entre 60 e 69 anos. A maior taxa foi observada no grupo de 70 a 79 anos (3.420,2/100 mil habitantes). Notou-se também uma redução inesperada na taxa de mortalidade no grupo com mais de 80 anos (4,1/100 mil habitantes) (Tabela 1).

A maioria dos óbitos ocorreu entre pessoas de raça/cor da pele branca (53,6%) e parda (32,4%). As maiores taxas de mortalidade foram observadas entre pretos (200/100 mil habitantes) e brancos (199/100 mil habitantes). A maior proporção de óbitos foi registrada ainda entre pessoas com 1 a 3 anos de escolaridade (23,4%) e entre aquelas com escolaridade ignorada (23,3%). Observou-se uma relação inversa entre escolaridade e mortalidade: indivíduos com 1 a 3 anos de estudo apresentaram uma taxa de 415/ 100 mil habitantes, enquanto aqueles com 12 anos ou mais apresentaram a menor taxa (21,2/100 mil habitantes) (Tabela 1).

A taxa de mortalidade média por DCV no Brasil, entre 2000 e 2023, foi equivalente a 169 óbitos/100 mil habitantes, com média anual de 337.553 mortes. A maior proporção de óbitos ocorreu na região Sudeste, que concentrou 47,4% dos registros, seguida pelas regiões Nordeste (25,4%) e Sul (15,8%). As taxas de mortalidade padronizadas variaram consideravelmente entre as regiões. As maiores taxas foram registradas no Sudeste (190/100 mil habitantes) e no Sul (187/100 mil habitantes). A menor taxa foi observada na região Norte, com 96 óbitos por 100 mil habitantes (Tabela 2).

Observou-se uma estabilidade relativa nas taxas de mortalidade por DCV no Brasil e por regiões ao longo do tempo, com variações sutis entre os anos. O gráfico reforçou também a permanência de altas taxas nas regiões Sul e Sudeste. A região Nordeste apresentou tendência de crescimento moderado nas taxas entre 2003 e 2015, com posterior estabilização. A região Centro-Oeste teve padrão semelhante, porém com taxas ligeiramente inferiores. A região Norte apresentou as menores taxas de mortalidade por DCV ao longo de todo o período analisado (Figura 1). 

**Tabela 1:** Caracterização sociodemográfica e taxa de mortalidade padronizada por 100 mil habitantes para doenças cardiovasculares. Brasil, 2003-2023 (n=7.088.623)

Variável	n (%)	Taxa de mortalidade
Sexo		
Masculino	3.721.949 (52,5)	181,9
Feminino	3.365.931 (47,4)	157,4
Ignorado	743 (0,1)	
Faixa etária (anos)		
<4	3.322 (0,1)	1,0
5-9	5.789 (0,1)	1,6
10-14	13.262 (0,1)	3,6
15-19	53.819 (0,7)	7,5
20-29	147.449 (2,0)	22,0
30-39	412.471 (5,8)	75,5
40-49	854.729 (12,0)	199,0
50-59	1.375.999 (19,4)	510,7
60-69	1.819.388 (25,6)	1.243,2
70-79	2.383.324 (33,6)	3.420,2
>80	6.374 (0,1)	4,1
Ignorada	12.697 (0,1)	1,0
Raça/cor da pele		
Branca	3.801.491 (53,6)	199,7
Preta	605.667 (8,5)	200,9
Amarela	42.541 (0,6)	96,2
Parda	2.299.138 (32,4)	132,1
Indígena	12.290 (0,1)	71,2
Ignorado	327.496 (4,6)	
Escolaridade (anos)		
Nenhuma	1.360.522 (19,1)	137,8
1-3	1.659.502 (23,4)	415,9
4-7	1.348.108 (19,0)	168,9
8-11	767.902 (10,8)	85,0
12 ou mais	299.363 (4,2)	21,2
Ignorado	1.653.226 (23,3)	

**Tabela 2:** Número, média de óbitos e taxa de mortalidade padronizada por 100 mil habitantes para doenças cardiovasculares por região. Brasil, 2003-2023 (n=7.088.623)

Região	n (%)	Média de óbitos	Taxa de mortalidade
Norte	342.127 (4,8)	16.292	96,9
Nordeste	1.807.161 (25,4)	86.055	157,6
Sudeste	3.365.596 (47,4)	160.266	190,1
Sul	1.124.748 (15,8)	53.559	187,5
Centro-Oeste	448.991 (6,3)	21.381	142,6
Brasil	7.088.623 (100,0)	337.553	169,4

**Figura 1:** Tendência da mortalidade por doenças cardiovasculares por regiões. Brasil, 2003-2023 (n=7.088.623)

A análise por pontos de inflexão no cenário nacional identificou dois segmentos distintos de tendência. Entre 2003 e 2008, observou-se crescimento anual de 1,97% (IC95% 1,19; 3,77). Esse aumento foi seguido por um período de estacionariedade, até que, entre 2020 e 2023, ocorreu novo incremento, de 2,34% ao ano (IC95% 0,97; 4,34). Nas regiões Norte e Nordeste, verificou-se padrão semelhante, com crescimento concentrado na série histórica. No Norte, a taxa aumentou 4,38% ao ano (IC95% 3,32; 7,02) entre 2003 e 2008, enquanto no Nordeste o incremento foi de 8,15% ao ano (IC95% 6,24; 10,69) de 2003 a 2007. Após esses períodos iniciais, a tendência tornou-se estacionária em ambas as regiões. Nas demais, o crescimento ocorreu no período mais recente. No Sudeste, registrou-se aumento anual de 2,16% (IC95% 0,57; 4,32) entre 2020 e 2023. No Sul, a elevação foi de 2,95% ao ano (IC95% 1,21; 6,76) entre 2019 e 2023. No Centro-Oeste, observou-se incremento anual de 2,38% (IC95% 0,89; 4,93) no intervalo de 2019 a 2023 (Tabela 3).

Na análise espacial do evento, obteve-se índice de Moran global de 0,02 (p-valor<0,05), o que indicou uma autocorrelação espacial fraca, porém positiva. Esse valor sugeriu que, embora tenha havido certa tendência de agrupamento de municípios com taxas semelhantes, essa associação foi pouco expressiva em escala nacional. Tal resultado foi coerente com a natureza heterogênea e disseminada do evento, já que as DCV representam a principal causa de morte no Brasil e afetam diferentes contextos geográficos, populacionais e socioeconômicos.

Essa interpretação foi reforçada pela comparação entre os mapas das taxas brutas e das taxas suavizadas de mortalidade. O mapa das taxas brutas (Figura 2A) evidenciou uma elevada variabilidade entre os municípios, com aparente concentração de valores elevados no Centro-Oeste, sobretudo em Mato Grosso e em Goiás. Essa distribuição bruta, contudo, é sensível a flutuações aleatórias, principalmente em localidades com pequena população, o que pode superestimar ou subestimar o risco real. Já o mapa com taxas suavizadas (Figura 2B) apresentou uma configuração espacial mais homogênea e epidemiologicamente consistente, revelou de forma mais clara os agrupamentos regionais. Mesmo após a suavização, observou-se persistência de áreas com elevada mortalidade nos dois estados supracitados, o que reforçou que eles correspondem a zonas de concentração da carga de mortalidade por DCV.

**Tabela 3:** Variação percentual anual (APC) e intervalos de confiança de 95% (IC95%) da mortalidade por doenças cardiovasculares por região. Brasil, 2003-2023 (n=7.088.623)

Região	Período	APC (IC95%)	p-valor	Tendência
Norte	2003-2008 2008-2023	4,38 (3,32; 7,02) 2,33 (2,03; 2,54)	<0,001 0,007	Crescente Crescente
Nordeste	2003-2007 2007-2023	8,15 (6,24; 10,69) 1,11 (0,92; 1,25)	<0,001 <0,001	Crescente Crescente
Sudeste	2003-2020 2020-2023	0,40 (0,08; 0,55) 2,16 (0,57; 4,32)	0,065 0,004	Estacionária Crescente
Sul	2003-2016 2016-2019 2019-2023	0,12 (-0,13; 2,65) -2,06 (-3,42; -0,17) 2,95 (1,21; 6,76)	0,216 0,024 0,008	Estacionária Decrescente Crescente
Centro-Oeste	2003-2019 2019-2023	0,47 (-0,11; 0,67) 2,38 (0,89; 4,93)	0,077 0,001	Estacionária Crescente
Brasil	2003-2008 2008-2020 2020-2023	1,97 (1,19; 3,77) 0,48 (-0,57; 0,67) 2,34 (0,97; 4,34)	0,003 0,157 <0,001	Crescente Estacionária Crescente

**Figura 2:** Distribuição das taxas brutas (A) e das taxas suavizadas (B) e identificação de aglomerados espaciais de alto e de baixo risco para mortalidade por doenças cardiovasculares pelo índice de Moran local (C) e de Getis-Ord Gi. Brasil, 2003-2023 (n=7.088.623)

A análise local de autocorrelação espacial permitiu identificar padrões geográficos da mortalidade por DCV no Brasil. O primeiro mapa (Figura 2C) ilustrou agrupamentos estatisticamente significativos de municípios com altas taxas, rodeados por municípios com taxas igualmente altas. Esses agrupamentos estão concentrados principalmente no sul do Mato Grosso e em Goiás. Já agrupamentos baixo-baixo concentraram-se majoritariamente na região Norte do Brasil e em alguns estados do Nordeste, como Maranhão e Bahia. 

Foram identificados ainda padrões de transição ou de dissonância espacial, como os agrupamentos alto-baixo, em Mato Grosso e em Goiás, e baixo-alto, em Mato Grosso e no Rio Grande do Sul, que representam áreas onde municípios com alta (ou baixa) mortalidade estão cercados por vizinhos com padrões opostos (Figura 2D). O mapa resume esses achados com uma abordagem binária por significância local (Getis-Ord Gi) e classificou os municípios em áreas quentes e áreas frias. As áreas quentes se concentraram nas mesmas regiões anteriormente destacadas como alto-alto, com grande densidade no Centro-Oeste. Já as áreas frias, que indicaram agrupamentos estatisticamente significativos de baixa mortalidade, foram amplamente distribuídas na região Norte, no litoral do Nordeste e na região Sul do país. 

## Discussão 

Os achados deste estudo evidenciam que a mortalidade por DCV no Brasil permanece elevada e distribuída de forma heterogênea entre grupos populacionais e territórios. Observou-se maior concentração do desfecho em segmentos socialmente vulneráveis, o que refletiu desigualdades estruturais que atravessam sexo, idade e escolaridade. Regionalmente, persistem contrastes marcantes, com estabilização relativa em áreas historicamente mais afetadas e crescimento progressivo em regiões antes menos impactadas, o que indicou uma redistribuição da carga da doença no país. A análise espacial revelou padrões geográficos que destacaram núcleos de maior risco e áreas de menor mortalidade, o que sugeriu que fatores socioeconômicos, estruturais e de organização dos serviços de saúde interagem de maneira distinta nos diferentes territórios. Esses resultados, em conjunto, reforçaram que a mortalidade por doença cardiovascular no Brasil é influenciada por determinantes sociais e contextuais e que sua distribuição atual expressa um cenário complexo de transição epidemiológica e de desigualdades persistentes.

Entre as limitações deste estudo, destaca-se a possibilidade de subnotificação ou de classificação inadequada das causas básicas de morte, especialmente em municípios com menor capacidade diagnóstica e cobertura de serviços. É importante também considerar que a cobertura do SIM apresenta variações regionais historicamente documentadas, o que inclui diferenças no sub-registro de óbitos, proporção desigual de causas mal definidas e heterogeneidade na completude dos campos de declarações de óbito. Essas variações podem influenciar a distribuição espacial observada da mortalidade por DCV, que devem ser interpretadas como partes do contexto que molda os achados. Por utilizar dados agregados, não foi possível controlar fatores individuais ou contextuais que poderiam explicar diferenças locais, o que implicou risco de falácia ecológica. Estudos futuros podem aprofundar fatores territoriais associados aos aglomerados identificados e explorar tendências em subgrupos populacionais específicos.

Neste estudo, observou-se maior risco de mortalidade por DCV entre homens, achado que acompanha resultados de autores nacionais ^13^, que identificaram maior vulnerabilidade masculina às DCV a partir de estudo epidemiológico baseado em dados agregados de mortalidade. Essa diferença pode ser compreendida pela interação entre fatores biológicos, como a menor proteção hormonal antes da menopausa nas mulheres, e fatores comportamentais, como maior prevalência de tabagismo entre homens, de uso abusivo de álcool e de dieta inadequada, além de menor adesão a práticas preventivas e ao acompanhamento em saúde, o que amplia o risco cardiovascular ao longo da vida ^14^.

A análise dos dados nesta pesquisa evidenciou elevação da mortalidade por DCV com o avanço da idade, padrão consistente com aqueles amplamente descritos na literatura ^15^. O envelhecimento intensifica a exposição acumulada a condições como hipertensão, diabetes e dislipidemias, além de levar à redução progressiva da reserva funcional cardiovascular e ao aumento da carga de comorbidades, elementos que se somam para explicar a maior vulnerabilidade dos idosos a esses desfechos. Curiosamente, constatou-se neste estudo menor taxa de mortalidade no grupo etário acima de 80 anos, achado inesperado que merece atenção. Esse resultado pode estar relacionado à subnotificação ou à classificação inadequada das causas básicas em idades avançadas, além do possível "efeito de sobreviventes", em que indivíduos que alcançaram maior longevidade tendem a apresentar perfil mais robusto e menor carga de fatores de risco. Esses aspectos podem explicar a discrepância observada e reforçam a necessidade de cautela ao interpretar taxas em faixas etárias idosas ^16^^-^^17^.

Nos resultados, identificou-se maior mortalidade por DCV entre pessoas de raça/cor da pele preta, o que reforçou a influência dos determinantes sociais na distribuição das DCV. Esse padrão dialoga com uma coorte americana, realizada com 50.808 pessoas, que apontou piores condições socioeconômicas, menor acesso a serviços de saúde e maior exposição a ambientes e a práticas que elevam o risco cardiovascular entre populações pretas no Brasil ^16^. Ressalta-se ser importante reconhecer que a classificação por raça/cor da pele é baseada em autodeclaração e que pode não capturar plenamente experiências de discriminação, o que indica a necessidade de análises interseccionais mais aprofundadas.

Os achados deste estudo evidenciaram uma relação inversa entre escolaridade e mortalidade cardiovascular, padrão que segue a tendência apontada pela literatura internacional, que reconhece a escolaridade como marcador central de desigualdades em saúde em estudo populacional realizado entre 2003 e 2019 ^18^. Uma menor escolaridade tende a estar associada a menos acesso à informação, a recursos, a serviços e a ambientes saudáveis, o que explica a maior mortalidade observada entre pessoas com até três anos de escolaridade e reforça a importância de estratégias educativas e de promoção da saúde sensíveis ao nível de educação da população ^19^.

No Brasil, os padrões regionais de mortalidade por DCV identificados neste estudo, especialmente o crescimento em regiões historicamente menos afetadas, evidenciaram semelhanças com países de renda média da Ásia e mostraram que a mortalidade por DCV tende a ser mais elevada em contextos com sistemas de saúde fragmentados, baixa cobertura de atenção primária à saúde e maior concentração de determinantes sociais adversos, características que também se refletem em parte do cenário brasileiro ^20^. 

Em contraste com os achados deste estudo, que evidenciaram crescimento da mortalidade por DCV em determinadas regiões brasileiras, países de renda média e de alta que avançaram em políticas públicas de prevenção, de controle do tabagismo, de ampliação de acesso a medicamentos cardiovasculares essenciais e de fortalecimento da atenção primária à saúde demonstraram reduções sustentadas na mortalidade por DCV nas últimas décadas ^21^^-^^22^. 

Neste estudo, a análise temporal revelou estabilização ou leve redução das taxas de mortalidade por DCV no Sudeste e no Sul do Brasil, em contraste com o crescimento contínuo observado no Norte e no Nordeste ao longo do período analisado. Essa divergência pode estar relacionada a diferenças estruturais e de acesso aos serviços de saúde, bem como ao avanço das políticas de prevenção e de manejo dos fatores de risco nas regiões mais desenvolvidas ^4^^,^^18^. O aumento das taxas de mortalidade nas regiões menos favorecidas também pode refletir mudanças comportamentais e ambientais, além de desigualdades persistentes no acesso a cuidados oportunos.

O crescimento da mortalidade cardiovascular observado nas regiões Norte e Nordeste pode ser influenciado por transformações demográficas aceleradas, urbanização heterogênea e transição nutricional, somadas ao aprimoramento progressivo dos sistemas de informação em saúde nesses territórios. Mas embora melhorias no registro possam ampliar a sensibilidade para identificar causas básicas cardiovasculares, o padrão crescente é compatível com expansão real da carga da doença ^23^^-^^24^.

Nos resultados deste estudo, a análise espacial revelou uma distribuição territorial heterogênea da mortalidade por DCV, com agregados de maior mortalidade especialmente em municípios de Mato Grosso e de Goiás. O padrão espacial identificado não ocorre de forma isolada: achados semelhantes foram descritos em estudo ecológico nacional que analisou a mortalidade por doenças cerebrovasculares no Brasil no período 1996-2015 e identificou maior risco em áreas com pior desenvolvimento humano e maior vulnerabilidade social ^25^. De forma convergente, estudo observacional com dados agregados do primeiro ano da pandemia de covid-19 apontou desigualdades regionais marcantes na mortalidade por DCV, associadas à capacidade assistencial e às disparidades estruturais dos sistemas locais de saúde ^26^. Em conjunto, essas evidências reforçaram que desigualdades socioeconômicas, cobertura assistencial irregular e diferenças na infraestrutura de saúde configuram microterritórios de maior risco cardiovascular, o que influenciou a distribuição dos óbitos.

Goiás e Mato Grosso têm vivenciado transformações socioespaciais intensas nas últimas décadas, impulsionadas pelo agronegócio, pelo crescimento de cidades médias e por fluxos migratórios expressivos. Esse processo de expansão econômica gera aumento rápido da urbanização e, comumente, mudanças no modo de vida local, com maior consumo de alimentos ultraprocessados, redução da atividade física e crescimento dos fatores de risco cardiometabólicos ^27^. Esses perfis têm sido observados, sobretudo, em municípios com forte presença do setor agroindustrial, onde a maior renda média não se traduz necessariamente em melhoria proporcional dos indicadores de saúde ^28^.

Somado a isso, Goiás e Mato Grosso apresentam grande heterogeneidade interna. Em Goiás, há contrastes marcantes entre a macrorregião de saúde de Goiânia, com melhor estrutura assistencial, e regiões como o norte e o nordeste goiano, onde persistem vazios sanitários, longas distâncias para serviços especializados e menor densidade de profissionais de saúde. Em Mato Grosso, a extensa dimensão territorial e a dispersão populacional agravam desafios na organização da assistência: muitos municípios dependem de polos regionais como Cuiabá, Sinop e Rondonópolis, o que gera atrasos importantes na linha de cuidado ^29^^-^^31^.

Esses estados também apresentam cobertura heterogênea da atenção primária à saúde. Em áreas rurais e periurbanas, são frequentes as fragilidades na Estratégia Saúde da Família, a rotatividade profissional, a dificuldade de continuidade do cuidado e a baixa capacidade de manejo das condições crônicas, especialmente hipertensão e diabetes, que são precursores diretos de complicações cardiovasculares ^32^. 

Somam-se ainda desigualdades sociais persistentes, com bolsões de pobreza em áreas de expansão agrícola, de comunidades tradicionais e de assentamentos rurais, onde o acesso aos serviços de saúde é mais limitado. Esse conjunto de fatores - transição epidemiológica acelerada, mudanças nos estilos de vida, heterogeneidades territoriais no acesso à saúde, dependência de polos regionais e fragilidade da atenção primária à saúde - oferece hipóteses plausíveis para explicar a maior concentração de mortalidade por DCV nos municípios de Mato Grosso e de Goiás, tal qual evidenciado no estudo ecológico nacional realizado em 2022 com dados do DATASUS, que relacionou doenças do sistema circulatório e determinantes sociais com foco na vulnerabilidade ^33^.

Diante das desigualdades regionais identificadas, a ampliação e a qualificação da atenção primária à saúde no território se faz uma estratégia central, com melhoria da capacidade diagnóstica e implementação de linhas de cuidado para hipertensão, diabetes e doença aterosclerótica. Em paralelo, é necessário aprimorar o acesso a serviços especializados por meio da regionalização da atenção cardiovascular, além da integração efetiva entre os níveis assistenciais, para garantir continuidade do cuidado. Medidas estruturantes, como vigilância ativa de fatores de risco, programas de promoção da saúde baseados em evidências e redução das desigualdades sociais que modulam o risco cardiovascular, também são fundamentais para reduzir diferenças interestaduais e intrarregionais. Esses caminhos apontam para a necessidade de políticas coordenadas que articulem prevenção, cuidado longitudinal e equidade na oferta de serviços ^34^. 

Este estudo demonstrou que a mortalidade por DCV no Brasil permanece elevada e que se concentra principalmente entre homens, idosos e indivíduos com baixa escolaridade. Observou-se tendência crescente nas regiões Norte, Nordeste e Centro-Oeste, além de aglomerados espaciais de alta mortalidade em municípios de Mato Grosso e de Goiás. Esses resultados evidenciaram desigualdades sociodemográficas e territoriais que devem orientar ações de vigilância e de planejamento em saúde.

## Data Availability

Os bancos de dados utilizados para análise estão disponíveis no repositório SciELO Data: https://doi.org/10.48331/SCIELODATA.MKZUPS.
